# Implementing Culture of Care in Germany

**DOI:** 10.3390/ani15192918

**Published:** 2025-10-08

**Authors:** Katharina Ameli, Stephanie Krämer

**Affiliations:** Interdisciplinary Centre for 3Rs in Animal Research (ICAR3R), Justus-Liebig-University, 35392 Giessen, Germany; stephanie.kraemer@vetmed.uni-giessen.de

**Keywords:** animals used for scientific purposes, animal welfare, human–animal studies, communication, animal agency

## Abstract

The implementation of the 3Rs principles is an essential part of daily routines and structural processes in animal research. The work of Russell and Burch regarding the 3Rs has been implemented in the field over the course of decades, but since 2002, the concept of a Culture of Care has come more to the forefront. In the present project, 503 experts in Germany were exploratively surveyed about their individual perceptions of Culture of Care and its implementation within their institutions. Using a questionnaire with closed questions (five-point agreement scale) and open questions, the data offer insights into Culture of Care in Germany. The results allow for the initial conclusion that a Culture of Care has not been fully established yet. Further research is needed to address a holistic and critical interdisciplinary Culture of Care that focuses on the animals’ perspective in a transformative way.

## Simple Summary

The 3Rs principles are an integral part in an animal research period. The implementation of the 3Rs principles is an essential part of daily routines and structural processes in animal research. Since 2002, a Culture of Care has been gaining prominence. A survey of 503 experts in Germany shows that it has not been fully established. Individual institutional steps for implementation can be recommended. Further research is needed to address the concrete implementation in different countries and research fields.

## 1. Introduction

The implementation of the 3Rs principles is an essential part of daily routines and structural processes in animal research. In addition to the initiation of numerous projects in the field of 3Rs research in Germany (e.g., BMBF replacement methods in animal experiments, funding by the Federal Institute for Risk Assessment, Ursula Händel Animal Welfare Prize of the German Research Foundation), theoretical debates on the 3Rs have emerged in recent years. These include analyses of the concept of a Culture of Care [1,2,3,4,5].

This concept is reflected in the EU Directive 2010/63, even though it is not named specifically as a Culture of Care. It is therefore named as a climate of care.

In order to strengthen the protection of laboratory animals for scientific purposes, the requirements of EU Directive 2010/63 had to be implemented for all Member States of the European Union. As a consequence, Germany amended the Animal Welfare Act and also issued the Animal Welfare Experimental Animals Ordinance. In 2018, the implementation of the EU requirements was regularly reviewed, and Germany had to face the accusation of an inadequate implementation. Corresponding infringement proceedings were initiated [6]. In June 2021, the sixth law amending the German Animal Welfare Act came into force, the contents of which have been applied from January 2022 onward. This resulted in changes concerning the handling of and working with laboratory animals. Among other things, it was emphasized that the conditions under which laboratory animals are kept must be continuously improved. Additionally, the institutional animal welfare committee needed to be restructured. It was explicitly mentioned that people entrusted with the care of laboratory animals must be appointed to the committee. [7,8].

The act also frequently referenced the work “The Principles of Humane Experimental Technique” written by Russell and Burch in 1959. The instrument of the authors for implementing ‘humanity’ in research are the 3R principles (Replace, Reduce, and Refine). Replace describes the replacement of animal experiments with alternative procedures. If this is not possible, the minimum necessary number of laboratory animals should be used in the sense of Reduce. If animal testing must be performed, an improvement is necessary to verify that the used animals experience no pain, suffering or harm [9].

The 3Rs are the cornerstone of that book and are quoted often. A deeper look at the work of Russell and Burch partially highlights a rationalization of the 3Rs. Other parts of the book, including the recommendation for interdisciplinary collaboration, remains undiscovered.

Russell and Burch saw a fundamental objective of the work as interweaving the humanities and science. In regard to the ambivalence between humanity and inhumanity within their work, the close interdependence between philosophy and sociology was described as an essential criterion for successful and objective science [9].

So, while Russell and Burch did not use the term Culture of Care in their book, their thoughts and beliefs can be connected to the current concept. By looking at what was being unconsciously ignored in animal research, Russell and Burch sensitized for a recognition of potential subjective parameters in research [9]. They fostered in some ways a change in culture in animal research.

Culture of Care has its roots in the area of nursing, care, and health, with a focus on communication [10]. The term is used for cultural sensitivity care to build a high-quality relationship [11].

In comparison, Culture of Care in animal research was first named in 2002. The National Animal Ethics Advisory Committee in New Zealand developed Culture of Care guidelines, which paved the way for a deeper discussion about the term and its connection to organizational culture [12]. Although the initial version of the concept seemed very rudimentary, it focused particularly on institutional work processes, staff development, and animal welfare. Developing from there, the scientific community established a reflection of culture in animal science, which led to the point where Culture of Care could be described as an organizational development process in which all levels (management level, science level, regulation level, and care level) in an institution must be involved [8].

The Norecopa Culture of Care network describes the term Culture of Care as generally being used to indicate a commitment to improving animal welfare, scientific quality, care of the staff, and transparency for the stakeholders. Norecopa sees a main goal in promotion of individual mind-set and behavior that continuously and proactively foster a next level above the 3R [13]. Consequently, an appreciative working atmosphere and a motivational environment are considered essential for employee satisfaction in animal research. Integrated into this are communication processes (between humans and animals) that go beyond verbal communication and therefore include non-verbal and paraverbal language between humans and animals [14].

Critical thinking in relation to personal and institutional values, beliefs, and attitudes is understood as a relevant aspect to ensure transformation, self-organization, and professionalism for humans and animals [8,15,16].

Overall, Culture of Care is a (social) organizational development process, which offers a transformation of current routines and processes. A chance is characterized by a dialogical negotiation process and a re-conceptualization for all stakeholders involved in animal experimentation. All levels involved (management, science, regulatory, and care) are involved as multipliers in the implementation of the 3Rs concept in general and in the implementation of a Culture of Care in particular [8,17]. Relevant empirical results of a recent explorative study give insights into the perspective of experts in levels of management, science, regulatory authorities, and care. The results illustrate individual types of knowledge about Culture of Care. This knowledge results from the decisions of an actor at the professional group and from the given legal and organizational structures [17]. This results in relevant (complex) categories that describe a Culture of Care, like organizational levels (e.g., 3R, communication, legal framework), biography/personality (e.g., routines, values, care identification), scientificity (research design, objectivity/subjectivity), and (animal) welfare (e.g., values, ethics, animal agency) [8]. Communication, appreciation of people and animals, as well as the attitude and professionalism are main categories for the implementation of a Culture of Care [18].

In regard to Russell and Burch [9], personality and social factors are main elements of a change in culture in animal experimentation. Addressing these individual habits of people working with animals and an analysis of the culturally legitimized routines that create inertia or rigidity have to be highlighted in the future [18].

Although the theoretical, empirical, and practical results offer a variety of characteristics encompassed within a Culture of Care, it has not yet been investigated how a Culture of Care is anchored in the structure of animal sciences. Based on these named findings, the current study aims to address this research gap.

By using a specific survey, different levels working in animal sciences were questioned in order to exploratively record the characteristics and implementation of a Culture of Care in Germany. The results identified indicators of understanding and implementation of a Culture of Care in Germany.

## 2. Materials and Methods

The survey was preceded by a detailed definition of a Culture of Care obtained through the meticulously theoretical analysis by Russell and Burch’s work and was underlined by an analysis of the theory of a Culture of Care. The in-depth analysis was important because the survey could only be formulated once all details relevant to the research objective had been determined in order to ensure the operationalization of the questionnaire [19]. The authors’ previous qualitative research [8] supported the generation of probabilistic hypotheses. However, such hypotheses do not claim to be lawful [19]. The formulation of the hypotheses as scientifically theory-based statements was based on the following two directions, with reference to the theory and the qualitative results: (1) Which aspects of a Culture of Care are known to professionals in animal research? and (2) Which aspects are already implemented in the structures of animal research?

The complexity of the elements of a Culture of Care allowed for including a variety of aspects and analyzing them in relation to the central research questions, named above. Using a Likert five-point agreement scale, where a high value means strong agreement and a low value means a strong disagreement, all questions were standardized and formulated as closed questions. This was intended to prevent the researchers from influencing the respondents [19]. The plausibility of the questions was checked in advance by a pre-test [19]. A total of 91 variables were collected.

The project collected and processed personal data. In addition to the ethical guidelines for conducting research projects of the German Sociological Association (DGS), the General Data Protection Regulation was used. No data were passed on to third parties, and all data were anonymized [19,20]. The project was ethically confirmed by the designated department of the authors’ institution. The questionnaire in German is added as File S1.

### 2.1. Sample

Due to the diverse distribution of skilled workers in different companies, a basic population in the form of structural data was not available. Therefore, the representativeness could not be quantified in terms of numbers [19]. No personalized invitations were possible as part of the exploratory approach. Subsequently, no structural equality between the population and the sample is given [19].

For this reason, the survey was based on a random sample (also known as an ad hoc sample). This sample space offers a non-probabilistic type of sampling and is particularly suitable for exploratory projects. A general call for participation was published through networks in the wider animal science community. This allowed motivated individuals interested in the topic to participate in the survey [21]. The response rate of the survey with fully completed questionnaires was n = 503.

### 2.2. Data Processing

Once the data collection was complete, the raw material was sorted, annotated, formatted, anonymized, and cleansed so that the cleansed and usable data sets could be used for further analysis [21]. The following criteria were taken into account: completeness, uniformity, exclusion of duplicate values, appropriate treatment of values, recognition of missing values, and the plausibility of response patterns [22]. It should be noted that the cleansing revealed that some participants who discontinued the survey at one point in time continued the survey at a later point in time. Only fully completed surveys were included [21]. In total, the data from n = 503 fully completed questionnaires were included in the analysis.

### 2.3. Data Evaluation and Data Analysis

The data preparation and statistical evaluation (frequency tables, diagrams, etc.) were conducted by using the statistical software SPSS and MS Excel. The cases were assigned to a group (management, science, regulatory, and care) based on the variable “profession”.

In the first step, particular attention was given to analyzing the mean values. In the second step, the standard deviations above 1.0 were also used. These offered insights into the spread of answers to individual questions. Consequently, the inhomogeneity of the answers was particularly integrated here as, according to our thesis, no clear, uniform opinion emerged, which in turn provided feedback on the implementation of the Culture of Care.

The qualitative responses from the questionnaire were evaluated using inductive categorization, clustered, and finally counted according to the frequency of mentions [23].

## 3. Results

The results have been presented descriptively. They focus on similarities and differences between the levels.

### 3.1. Demographic Data

**Figure 1:**
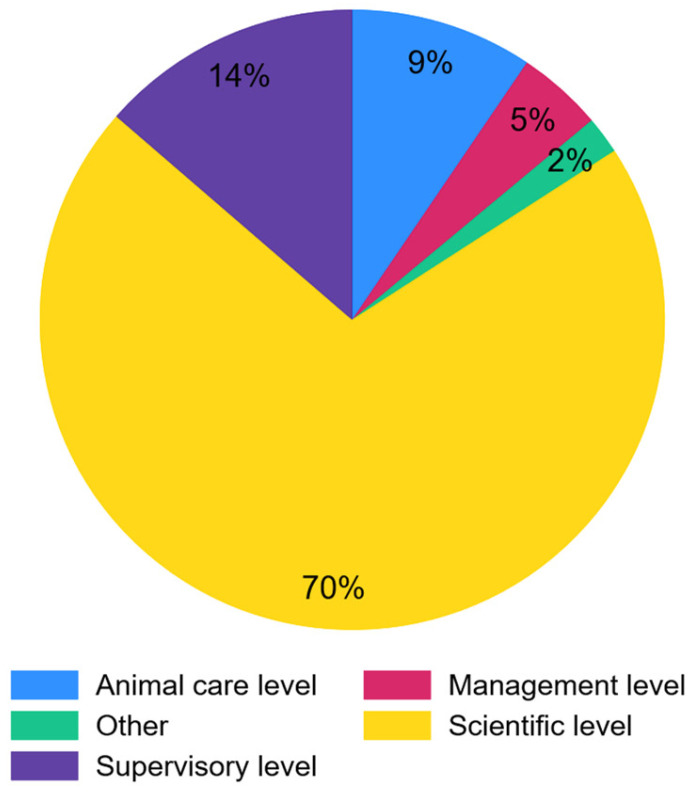
Demographic data.

The demographic data of the research are based on Figure 1. The science group accounts for the largest proportion of respondents at 70%. This is followed by the regulatory level with 14%, the care level with 9%, the management level with 5%, and others with 2%. Others were not integrated into the analysis due to working outside lab animal institutions. The mean age of all respondents was 39.99 years. A question regarding gender classification was not given. Of all the animal facilities in which the respondents work, 50.5% are externally accredited.

**Figure 2:**
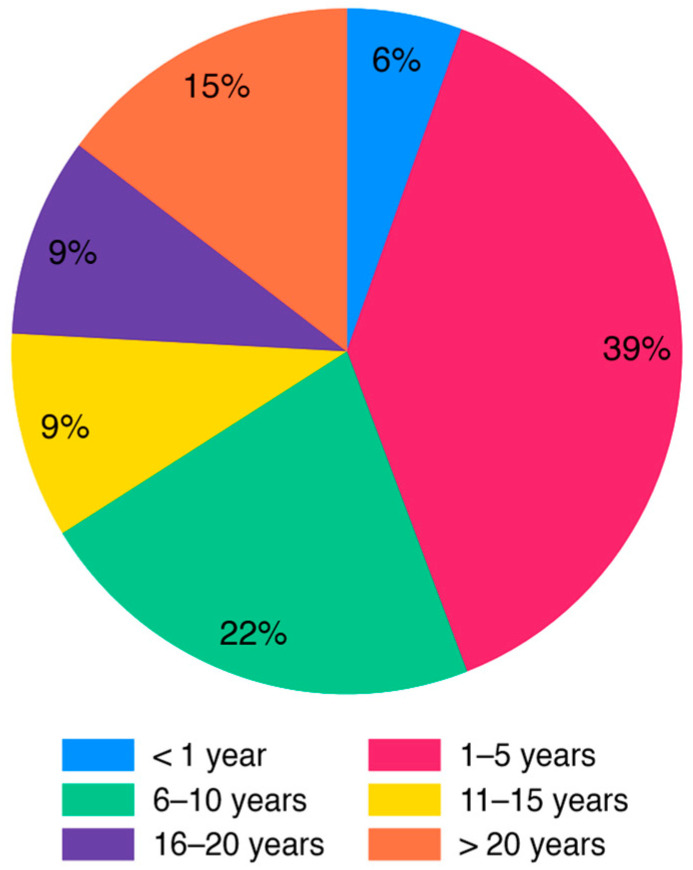
Duration of activity in animal research.

The respondents’ previous length of service varies, although the 1- to 5-year period of service accounts for the highest proportion (39%), as Figure 2 shows.

### 3.2. Fundamentals of the 3Rs

The majority of participants stated that they were familiar with the 3Rs principles and that they apply them in their institution (mean value 4.77 on the five-point agreement scale).

With regard to awareness of Russell and Burch’s work, this topic is familiar at the levels of management (mean 4.04 on Likert scale) and regulatory level (mean 3.94 on Likert scale, standard deviation 1.083), as well as the as the employers involved in animal care (mean 2.90 on Likert scale, standard deviation 1.322) and science level; however, it is only indicated at a medium level of agreement at both levels.

The implementation of existing internal guidelines for the implementation of the 3Rs shows that management level (mean 4.00 on Likert scale), care level (mean 4.19 on Likert scale), and science level (mean 4.19 on Likert scale) agree with the use of internal guidelines. The measures in these guidelines go beyond the minimum legal requirements.

The management level indicates with a high level of agreement that employees are encouraged to take time to reflect and make suggestions for improving animal welfare (mean value 4.39). However, this agreement differs in the care level (mean 3.67 on Likert scale, standard deviation 1.206), science group (mean 3.99 on Likert scale, standard deviation 1.062), and regulatory level (mean 4.2 on Likert scale, standard deviation 1.013) with a medium to high level of agreement and a high dispersion of responses.

### 3.3. Fundamentals of a Culture of Care

The qualitative data from one open question within the questionnaire asked which three aspects of the term Culture of Care spontaneously came to participants’ mind. By using inductive categorization, clustering, and counting, a total of 129 categories were formed inductively. They are introduced in Table 1.

**Chart 1:**
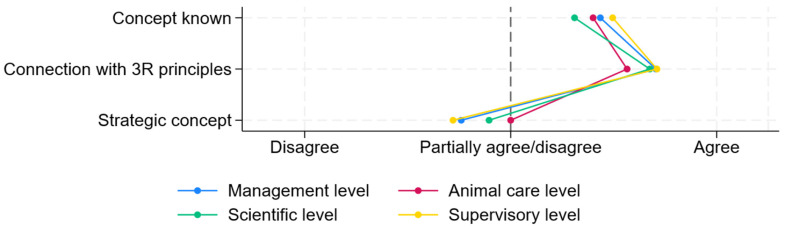
Concept of Culture of Care.

Further analysis shows the following results for the implementation of the Culture of Care: Table 2 and Chart 1 illustrates that the Culture of Care is not yet fully known at all levels and that strategic concepts are largely non-existent.

**Chart 2:**
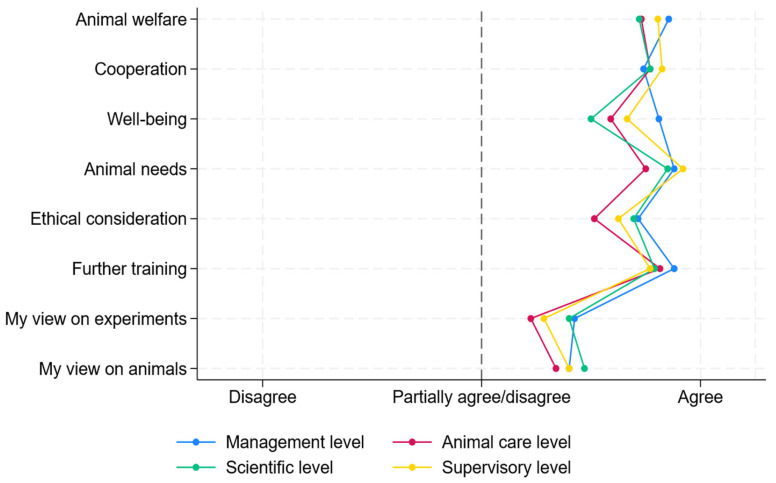
Significance of a Culture of Care.

Furthermore, a practiced Culture of Care is not seen as a tool to promote rejection of animal experimentation from animal experiments (care level: mean 2.95; science group: mean 3.01, standard deviation 1.023; management level: mean 2.91; regulatory level: mean 3.11, standard deviation 1.117). Table 3 and Chart 2 illustrates the perception of significance of a Culture of Care.

The results show high levels of agreement with regard to improving animal welfare and animal protection, ethical considerations, and the importance of human well-being. At the same time, it is clear that the personal perspective on the experiments and the view of animals show a largely medium level of agreement. This is characterized by a high standard deviation.

**Chart 3:**
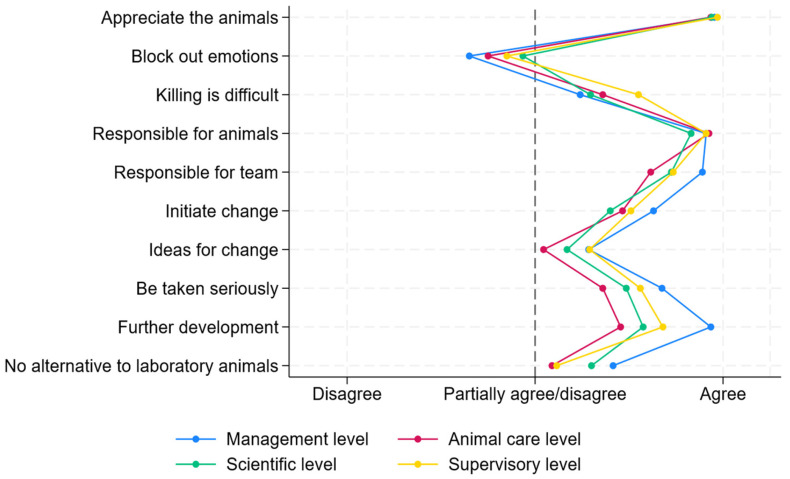
Individual perspectives in the interaction between animals and humans.

The participants’ individual attitudes towards animals and employees are shown in Table 4 and Chart 3, with a high level of appreciation and a sense of responsibility towards animals. However, the actual implementation here shows a low approval rating.

The responsibilities within and between the levels show that all levels ascribe mutual responsibility for animal welfare, with the regulatory level receiving least approval. It is closely connected to the responsibility for animal welfare, which Table 5 illustrates.

In comparison to the responsibility of the individual level for animals, the responsibility for the well-being of the employees rated for the management level with a high level of agreement. For the care, scientific, and regulatory levels, only a medium level of agreement is given for the responsibility for the well-being of the employees.

**Chart 4:**
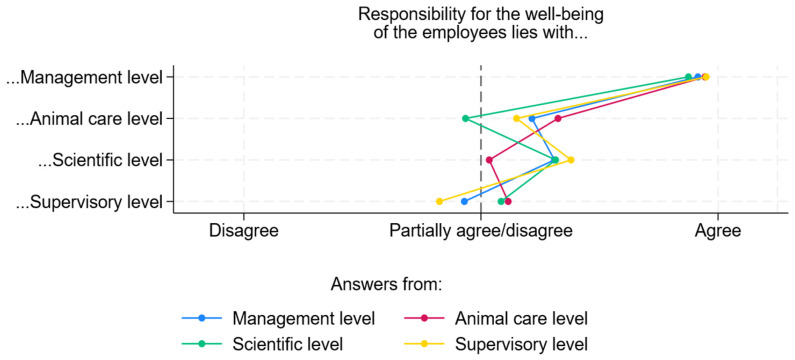
Responsibility for the well-being of employees.

This responsibility is illustrated in Table 6 and Chart 4.

**Chart 5:**
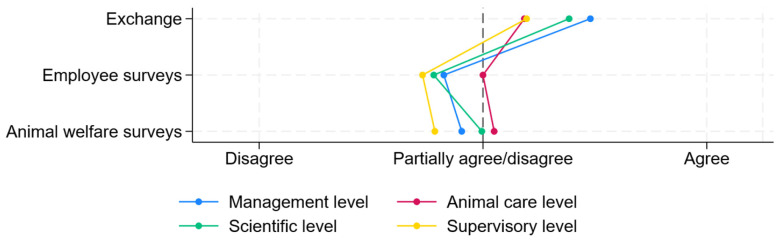
Communication and well-being.

Table 7 and Chart 5 illustrates that communication between the levels is largely rated as successful within and between the levels, with medium-to-high agreement. Nevertheless, it should be noted that there is a particularly high level of dispersion between the regulatory and the care level.

The exchange between the science and care level is at a medium level of approval. It should be emphasized that there are hardly any organization surveys on the well-being of animals and humans.

## 4. Discussion

The results allow for initial conclusions that a Culture of Care has not yet been fully established in Germany. While the majority of all respondents stated that they were personally familiar with the 3Rs principles, awareness of the content of Russell and Burch’s work was significantly low in all levels. It can be assumed that although the core essence of the 3Rs is known, there has been no differentiated consideration and reception on the work of the authors in question overall.

When it comes to authors’ work in general, this thesis is in line with the findings of Tannenbaum and Bennett (2015) [2]. The authors stated that a majority of researchers have hardly studied Russell and Burch’s work in any depth. They concluded that there is a discrepancy in the implementation of the 3Rs, as no differentiated approaches and application of the entire work can be found in everyday animal research.

Although there has been increased discussion in the context of a Culture of Care within publications and at conferences [13], a final implementation or a uniform consensus on what a Culture of Care is has not yet been conclusively established [15,16]. In conclusion, the lack of a generally valid and recognized definition of a Culture of Care can be identified as a stumbling block that makes implementation difficult to integrate into organizational processes. Although the concept is partially known, an integration or transformation of the culture has not taken place. The reasons for this can be seen in different perceptions of the Culture of Care and its specific task [17].

As a result, the explorative results from Germany show that the complexity of a Culture of Care has not yet been combined intensively enough with existing routines and work processes from a reflective perspective. This is particularly evident in the consideration of ideas, thoughts, and innovations from employees, which are underrepresented. Although the results show that employees are encouraged to take their time and reflect on this topic, the resulting processes are not visible in the sense of integration into organizational processes in the case of significant reflection and change processes. This shows that the approval of the consideration of personal perspectives and ideas for change regarding the effects of animal experiments on animals is only moderately positive.

Referring back to Table 3, it can be seen that ensuring animal welfare received the highest number of mentions. A provocative statement that arises from this is the extent to which there is a correlation between individual values and personality traits in the perception and evaluation of experiments and the concrete effects on animals.

The implementation of the Culture of Care is linked to the acceptance of change processes by each individual. In this context, however, the care level shows a difference compared to the other levels surveyed. For example, there is a discrepancy in the perception of expertise and personal development in the care level compared to the other levels surveyed. Consequently, the care level appears to be less integrated into change processes; however, it certainly has the strongest animal contact.

The question is underpinned by the results on communication within and outside the levels. For example, there are no comprehensive and regular surveys on animal welfare and/or employees, nor is the exchange in the form of concrete courses of action firmly established in the institutional structures. Nevertheless, communication is described as successful.

The conclusion itself suggests that the implementation of a Culture of Care is not yet integrated across the board in the institutions. In fact, organizational development towards a Culture of Care only appears to be practiced in isolated cases [24]. However, it should be noted that while some aspects of the Culture of Care are already being taken into account, essential animal perspectives, such as animal agency, are being highly ignored [17]. This is hardly surprising, as can also be seen in discussions at events on the topic. Perspectives from the humanities and social sciences are often seen as barely relevant. At a recent conference at Justus Liebig University, some researchers explicitly criticized the perspectives of these disciplines. Their arguments can be linked to critical animal studies and would emerge from an ideological bubble and hardly offer any pragmatic solutions [25].

However, a deeper look at the work of Russell and Burch (1959) reveals that the 3R concept requires precisely these perspectives [9]. It is obvious that a holistic perspective can only be created through inter- and cross-disciplinary thinking and action. Simply referring to animal welfare while ignoring scientific and other perceived trends and theories seems to have little in common with the transformation of an existing culture.

An additional aspect is also given in the high level of agreement in the appreciation of the animals, in which emotional involvement is also evident. When considering one’s own emotions in the context of work activities, it is clear that feelings and emotions are suppressed at all levels. The care level shows particular mechanisms for blocking out emotions, although the data on the killing of animals provide evidence that not all participants perceive this as a stressful activity.

The reasons for the respondents’ differing perceptions of stress and the consequences of ignoring human emotions for ensuring animal welfare and responsibility towards them remain unclear.

Responsibility for animals is an essential core element that underpins a Culture of Care. All levels refer to equal responsibility for animals.

## 5. Conclusions

Overall, the implementation of the 3Rs and the Culture of Care shows that relevant knowledge already exists, but this does not yet appear to have been translated into uniform practices.

The results do not allow for any conclusions to be drawn as to the extent to which the entire basic idea of the 3Rs concept has been implemented in the facilities with the entire depth and sophistication required of a holistic implementation of the 3Rs according to Russell and Burch. This is particularly important to discuss in light of the fact that Russell and Burch had already described an organizational development process in their work that is similar to the cornerstones of a Culture of Care and places a particular focus on the animal. However, this remains largely unconsidered in today’s reception of the work.

This finding is also consistent with the lack of implementation in the course of the criticism of the EU directive mentioned at the beginning. Indirectly, this represents a legal requirement. It can be assumed that an amendment to the law on these points would probably not have been enforced if the husbandry conditions and self-evident embedding of animal care staff in the legally required animal welfare committee had been credibly and comprehensibly mapped.

It can be assumed that the system in which animal research is located is currently still largely self-constructing and self-regulating and consequently maintains these structures [26]. Further research that approaches the topic in a holistic and critical interdisciplinary way is needed. First, (multispezies) ethnographic research may be a relevant changemaker, due to its social sciences research approach. Secondly, similar surveys in different countries and by will be helpful to highlight differences, exchange information and perceptions, and make a difference together. Animal Welfare, staff welfare, and research quality are inseparably linked in a Culture of Care. All three must be equivalent goals.

As a result, the following concrete institutional steps can be recommended for a culture that has not yet been well established:

1. Mission Statement
  - Management assumes official responsibility
  - Critical reflection on the current culture for humans and animals within the institution
  - Clarification of responsibilities and roles
2. Provide resources
  - Qualified personnel
  - Budgeting for further development
  - Further education and training related to culture of care
  - Openness, willingness, space, and time for a dialogue between levels
3. Develop an action plan for implementation
  - Mentoring
  - Lesson-learned culture with error tolerance
  - How to integrate animal’s agency

Overall, the implementation of a Culture of Care can only exist with an engagement from (intuitional) leaders and staff, a real animal participation, resources, further and critical education, and a real will for change.

## Figures and Tables

**Table 1:** Named categories within the Culture of Care paradygm.

Category	Frequency of Mention
Ensuring animal protection/animal welfare	233
3Rs (implementation and further development)	113
Refine(ment)	45
Responsibility	36
Training, continuing education, and further education/trained personnel	34
(Duty of) care for humans and animals	76
Respect	26
Alternatives/replacement methods	25
Communication	65
Reduce/reduction	25

**Table 2:** Concept of Culture of Care.

	Management Level			Animal Care Level			Scientific Level			Regulatory Level		
	N	M	SD	N	M	SD	N	M	SD	N	M	SD
I am familiar with the concept of a Culture of Care.	23	3.87	1.01	46	3.80	1.13	354	3.62	1.09	69	3.99	0.96
I see a direct connection between the 3Rs principles and the Culture of Care.	22	4.41	0.91	39	4.13	0.80	303	4.35	0.83	67	4.42	0.72
My institution is implementing a strategic concept for a Culture of Care.	21	2.52	1.08	37	3.00	1.05	259	2.79	1.20	62	2.44	1.15

Response scale: 1 = Strongly disagree to 5 = Strongly agree. N = Number, M = Arithmetic mean, SD = Standard deviation. Colours of each level are linked from table and chart.

**Table 3:** Significance of a Culture of Care.

	Management Level			Animal care Level			Scientific Level			Regulatory Level		
	N	M	SD	N	M	SD	N	M	SD	N	M	SD
…improvements in animal welfare.	21	4.71	0.56	39	4.46	0.60	294	4.44	0.73	64	4.61	0.68
…productive, communicative cooperation between all parties involved.	21	4.48	0.68	39	4.54	0.56	292	4.54	0.68	63	4.65	0.74
…human well-being.	21	4.62	0.67	39	4.18	0.82	284	4.00	1.03	63	4.33	0.86
…consideration of the needs of laboratory animals used in experiments.	21	4.76	0.54	38	4.50	0.69	295	4.70	0.51	63	4.84	0.37
…ethical consideration of the use of animals.	21	4.43	1.03	38	4.03	1.10	286	4.39	0.86	60	4.25	1.07
…continuous training of employees.	21	4.76	0.54	38	4.63	0.63	294	4.58	0.69	63	4.54	0.71
…that my personal view of the experiments is taken into account.	20	3.85	1.04	38	3.45	1.01	287	3.80	1.05	61	3.57	1.12
…that my personal view of the effects of animal testing on the laboratory animals is taken into account.	20	3.80	1.15	38	3.68	1.04	283	3.94	1.02	61	3.80	1.06

Response scale: 1 = Strongly disagree to 5 = Strongly agree. N = Number, M = Arithmetic mean, SD = Standard deviation. Colours of each level are linked from table and chart.

**Table 4:** Individual perspectives in the interaction between animals and humans.

	Management Level			Animal Care Level			Scientific Level			Regulatory Level		
	N	M	SD	N	M	SD	N	M	SD	N	M	SD
I appreciate the animals I work with.	23	4.91	0.29	46	4.87	0.40	350	4.88	0.36	66	4.94	0.24
I largely block my feelings and emotions while working with laboratory animals.	23	2.30	1.11	46	2.50	1.23	351	2.87	1.18	66	2.70	1.05
Killing animals bothers me.	23	3.48	0.99	47	3.72	1.19	345	3.59	1.14	63	4.10	0.98
I feel a special responsibility for animals.	22	4.82	0.40	47	4.85	0.42	351	4.66	0.60	67	4.82	0.39
I feel responsible for good teamwork.	23	4.78	0.52	47	4.23	0.70	347	4.45	0.78	66	4.47	0.64
I feel capable of initiating change.	23	4.26	0.69	46	3.93	1.06	350	3.80	1.08	66	4.02	1.03
When I contribute to ideas for change, they are implemented.	23	3.57	0.59	46	3.09	0.96	328	3.34	0.95	67	3.58	0.94
I am taken seriously as a person and for my knowledge.	23	4.35	0.65	47	3.72	1.12	343	3.97	0.98	67	4.12	0.93
My institution offers me opportunities for regular personal development.	23	4.87	0.34	47	3.91	1.21	350	4.15	0.96	66	4.36	0.94
There is no alternative to the use of laboratory animals for scientific progress.	23	3.83	0.98	45	3.18	0.78	349	3.60	1.04	69	3.23	1.07

Response scale: 1 = Strongly disagree to 5 = Strongly agree. N = Number, M = Arithmetic mean, SD = Standard deviation. Colours of each level are linked from table and chart.

**Table 5:** Responsibility for animal welfare.

The Responsibility for the Welfare of the Animals Lies with the…	Responding:											
	Management Level			Animal Care Level			Scientific Level			Regulatory Level		
	N	M	SD	N	M	SD	N	M	SD	N	M	SD
…Management Level	23	4.83	0.39	47	4.43	0.90	347	4.31	0.96	68	4.71	0.65
…Animal care Level	23	4.65	0.83	47	4.74	0.77	351	4.60	0.75	69	4.80	0.44
…Scientific Level	21	4.76	0.54	47	4.40	1.06	350	4.58	0.78	68	4.79	0.48
…Regulatory Level	22	4.14	1.08	46	4.15	0.99	347	4.08	1.05	69	4.07	1.10

Response scale: 1 = Strongly disagree to 5 = Strongly agree. N = Number, M = Arithmetic mean, SD = Standard deviation. Colours of each level are linked from table and chart.

**Table 6:** Responsibility for the well-being of employees.

Responsibility for the Well-Being of Employees Lies with the…	Responding:											
	Management Level			Animal Care Level			Scientific Level			Regulatory Level		
	N	M	SD	N	M	SD	N	M	SD	N	M	SD
…Management Level	23	4.83	0.49	45	4.89	0.38	345	4.75	0.68	69	4.90	0.35
…Animal Care Level	21	3.43	1.29	46	3.65	1.39	332	2.87	1.26	69	3.30	1.15
…Scientific Level	21	3.62	1.02	45	3.07	1.21	330	3.63	1.17	68	3.76	1.14
…Regulatory Level	21	2.86	1.49	44	3.23	1.33	332	3.17	1.33	68	2.65	1.43

Response scale: 1 = Strongly disagree to 5 = Strongly agree. N = Number, M = Arithmetic mean, SD = Standard deviation. Colours of each level are linked from table and chart.

**Table 7:** Communication and well-being.

	Management Level			Animal Care Level			Scientific Level			Regulatory Level		
	N	M	SD	N	M	SD	N	M	SD	N	M	SD
There is regular communication between scientists and animal keepers, during which they exchange information.	23	3.96	0.83	43	3.37	1.13	329	3.77	1.09	57	3.39	1.00
Regular surveys are conducted on the well-being of employees.	23	2.65	1.23	44	3.00	1.26	314	2.56	1.31	54	2.46	1.36
Regular surveys are conducted on the well-being of the animals.	21	2.81	1.33	42	3.10	1.32	312	2.99	1.42	53	2.57	1.25

Response scale: 1 = Strongly disagree to 5 = Strongly agree. N = Number, M = Arithmetic mean, SD = Standard deviation. Colours of each level are linked from table and chart.

## Data Availability

Data are unavailable due to privacy and ethical restrictions.

## Supplementary Materials

The following supporting information can be downloaded at https://www.mdpi.com/article/10.3390/ani15192918/s1. File S1: The original questionnaire in German is available.
