# Supplementary material for: Implementing Culture of Care in Germany
Source: Animals (Basel). 2025 Oct 8;15(19):2918. doi: 10.3390/ani15192918 (PMC12524104; doi:10.3390/ani15192918)
Supplement: Supplementary file 1 [file animals-15-02918-s001.zip › animals-3893899-supplementary.pdf]

Katharina Ameli & Stephanie Krämer
ICAR3R, Justus-Liebig-Universität Gießen

Culture of Care - Umfrage

Sehr geehrte Teilnehmende,

zunächst einmal möchten wir uns bedanken, dass Sie - trotz der derzeitigen Pandemiesituation - das Forschungsprojekt, gefördert durch die SET-Stiftung unterstützen.

Wir, das Interdisciplinary Centre for 3R's in Animal Research der Justus-Liebig-Universität begrüßen Sie herzlich zu dieser Umfrage.

Das Ziel der Umfrage ist es, die Bedeutung der Culture of Care (zu deutsch auch als Kultur der Fürsorge und Wertschätzung) in versuchstierkundlichen Einrichtungen in Deutschland und der Schweiz aus Ihrer ganz persönlichen Sicht zu erfassen.

Diese Umfrage richtet sich an alle Personen die im versuchstierkundlichen Bereich tätig sind (Tierhausleitung, Behördenvertreter*innen, Tierpflegende, Tierschutzbeauftragte, Wissenschaftler*innen etc.). Ihre Angaben helfen uns erstmalig differenzierte Angaben zur Bedeutung der Culture of Care in Deutschland und der Schweiz statistisch auszuwerten.

Der Schutz der Teilnehmenden an der Befragung ist uns ein hohes Anliegen. Daher werden alle Ihre Angaben selbstverständlich anonym und datenschutzkonform verarbeitet.

Die Umfrage wird ca. 15 Minuten in Anspruch nehmen.

Katharina Ameli & Stephanie Krämer
ICAR3R, Justus-Liebig-Universität Gießen

Demographische Angaben

**Beruf[1]:**	**Alter:**
**In welchem Institut/Forschungsgesellschaft sind Sie tätig?**	
**Funktion im Unternehmen:**	
**Dauer der Tätigkeit:**	
**Tierhaltung ist extern akkreditiert:** ☐ Ja ☐ Nein ☐ Keine Antwort	

Bitte geben Sie an, inwiefern Sie den folgenden Aussagen zustimmen.

Frageblock zu den 3R-Prinzipien

1. Die 3R-Prinzipien sind mir persönlich bekannt.

Stimme gar nicht zu	Stimme eher nicht zu	Teils, teils	Stimme eher zu	Stimme voll zu	Keine Antwort
☐	☐	☐	☐	☐	☐

2. Die 3R-Prinzipien finden in meiner Institution Anwendung.[2]

Stimme gar nicht zu	Stimme eher nicht zu	Teils, teils	Stimme eher zu	Stimme voll zu	Keine Antwort
☐	☐	☐	☐	☐	☐

3. Das Werk von Russell und Burch „The Principles of Humane Experimental Technique“ kenne ich gut.

Stimme gar nicht zu	Stimme eher nicht zu	Teils, teils	Stimme eher zu	Stimme voll zu	Keine Antwort
☐	☐	☐	☐	☐	☐

[1] Pflichtangabe
[2] Pflichtangabe

Katharina Ameli & Stephanie Krämer
ICAR3R, Justus-Liebig-Universität Gießen

Frageblock zur Umsetzung der 3R-Prinzipien und einer Culture of Care

Hinweis: Fragen erscheinen nur, falls Frage 2 mit *„teils, teils“*, *„stimme eher zu“* oder *„stimme voll zu“* beantwortet wurde.

4. Existierende, interne Leitlinien zur Gewährleistung der 3R werden angewendet.

Stimme gar nicht zu	Stimme eher nicht zu	Teils, teils	Stimme eher zu	Stimme voll zu	Keine Antwort
☐	☐	☐	☐	☐	☐

5. Maßnahmen, welche über die gesetzlichen Mindestanforderungen im Umgang mit Versuchstieren hinaus gehen, werden angewendet.

Stimme gar nicht zu	Stimme eher nicht zu	Teils, teils	Stimme eher zu	Stimme voll zu	Keine Antwort
☐	☐	☐	☐	☐	☐

6. Mitarbeiter*innen werden zum Innehalten, Nachdenken und Vorschlägen zu Verbesserungen des Tierschutzes ermutigt.

Stimme gar nicht zu	Stimme eher nicht zu	Teils, teils	Stimme eher zu	Stimme voll zu	Keine Antwort
☐	☐	☐	☐	☐	☐

7. Die national oder behördlich geforderten versuchstierkundlichen Weiterbildungen werden durchgeführt.

Stimme gar nicht zu	Stimme eher nicht zu	Teils, teils	Stimme eher zu	Stimme voll zu	Keine Antwort
☐	☐	☐	☐	☐	☐

8. Falls ja, wo finden diese Weiterbildungen statt?

Intern	Extern	Sowohl als auch	Keine Antwort
☐	☐	☐	☐

9. Es existiert ein regelmäßiger Austausch, bei denen sich Wissenschaftler*innen und Tierpfleger*innen gegenseitig informieren.

Stimme gar nicht zu	Stimme eher nicht zu	Teils, teils	Stimme eher zu	Stimme voll zu	Keine Antwort
☐	☐	☐	☐	☐	☐

10. Eine konstruktive und offene Interaktion mit zuständigen Behörden wird in unserer Institution praktiziert.

Stimme gar nicht zu	Stimme eher nicht zu	Teils, teils	Stimme eher zu	Stimme voll zu	Keine Antwort
☐	☐	☐	☐	☐	☐

11. Ein interner Tierschutzausschuss zur kritischen Diskussion von (u.a. schwer belasteten) Tierversuchsvorhaben existiert in meiner Institution.

Stimme gar nicht zu	Stimme eher nicht zu	Teils, teils	Stimme eher zu	Stimme voll zu	Keine Antwort
☐	☐	☐	☐	☐	☐

12. Die Möglichkeit, Bedenken an eine Vertrauensperson zu richten, ist gegeben.

Wenn ja, welche Funktion hat die Vertrauensperson?
____

Stimme gar nicht zu	Stimme eher nicht zu	Teils, teils	Stimme eher zu	Stimme voll zu	Keine Antwort
☐	☐	☐	☐	☐	☐

13. Befragungen zum Wohlbefinden von Mitarbeiter*innen werden regelmäßig durchgeführt.

Stimme gar nicht zu	Stimme eher nicht zu	Teils, teils	Stimme eher zu	Stimme voll zu	Keine Antwort
☐	☐	☐	☐	☐	☐

14. Befragungen von Mitarbeiter*innen zum Wohlbefinden von Tieren werden regelmäßig durchgeführt.

Stimme gar nicht zu	Stimme eher nicht zu	Teils, teils	Stimme eher zu	Stimme voll zu	Keine Antwort
☐	☐	☐	☐	☐	☐

Frageblock zum Konzept der Culture of Care I

15. Das Konzept einer Culture of Care ist mir bekannt.[3]

Stimme gar nicht zu	Stimme eher nicht zu	Teils, teils	Stimme eher zu	Stimme voll zu	Keine Antwort
☐	☐	☐	☐	☐	☐

16. In unserer Organisation ist mir eine interne Leitlinie zum ethischen Tierschutz bekannt.

Stimme gar nicht zu	Stimme eher nicht zu	Teils, teils	Stimme eher zu	Stimme voll zu	Keine Antwort
☐	☐	☐	☐	☐	☐

17. Ich sehe einen direkten Zusammenhang zwischen den 3R-Prinzipien und dem Konzept einer Culture of Care.

Stimme gar nicht zu	Stimme eher nicht zu	Teils, teils	Stimme eher zu	Stimme voll zu	Keine Antwort
☐	☐	☐	☐	☐	☐

18. Ein strategisches Konzept zur Implementierung einer Culture of Care in meiner Institution wird umgesetzt.

Stimme gar nicht zu	Stimme eher nicht zu	Teils, teils	Stimme eher zu	Stimme voll zu	Keine Antwort
☐	☐	☐	☐	☐	☐

[3] Pflichtangabe

Frageblock zum Konzept der Culture of Care II

Hinweis: Fragen erscheinen nur, falls Frage 15 mit *„teils, teils"*, *„stimme eher zu"* oder *„stimme voll zu"* beantwortet wurde.

19. Culture of Care ist für mich eine Verbesserung des Tierschutzes.

Stimme gar nicht zu	Stimme eher nicht zu	Teils, teils	Stimme eher zu	Stimme voll zu	Keine Antwort
☐	☐	☐	☐	☐	☐

20. Culture of Care bedeutet für mich eine produktive, kommunikative Zusammenarbeit zwischen den beteiligten Personen.

Stimme gar nicht zu	Stimme eher nicht zu	Teils, teils	Stimme eher zu	Stimme voll zu	Keine Antwort
☐	☐	☐	☐	☐	☐

21. Culture of Care bedeutet für mich menschliches Wohlbefinden.

Stimme gar nicht zu	Stimme eher nicht zu	Teils, teils	Stimme eher zu	Stimme voll zu	Keine Antwort
☐	☐	☐	☐	☐	☐

22. Culture of Care bedeutet für mich die Berücksichtigung der Bedarfe und die Bedürfnisse eingesetzter Versuchstiere.

Stimme gar nicht zu	Stimme eher nicht zu	Teils, teils	Stimme eher zu	Stimme voll zu	Keine Antwort
☐	☐	☐	☐	☐	☐

23. Culture of Care ist für mich eine ethische Abwägung zum Einsatz von Tieren.

Stimme gar nicht zu	Stimme eher nicht zu	Teils, teils	Stimme eher zu	Stimme voll zu	Keine Antwort
☐	☐	☐	☐	☐	☐

24. Culture of Care bedeutet für mich kontinuierliche Fortbildung von Mitarbeiter*innen.

Stimme gar nicht zu	Stimme eher nicht zu	Teils, teils	Stimme eher zu	Stimme voll zu	Keine Antwort
☐	☐	☐	☐	☐	☐

25. Culture of Care bedeutet für mich, dass meine persönliche Sicht auf die durchgeführten Versuche berücksichtigt wird.

Stimme gar nicht zu	Stimme eher nicht zu	Teils, teils	Stimme eher zu	Stimme voll zu	Keine Antwort
☐	☐	☐	☐	☐	☐

26. Culture of Care bedeutet für mich, dass meine persönliche Sicht auf die Auswirkungen auf die Versuchstiere berücksichtigt werden.

Stimme gar nicht zu	Stimme eher nicht zu	Teils, teils	Stimme eher zu	Stimme voll zu	Keine Antwort
☐	☐	☐	☐	☐	☐

27. Für die Umsetzung der Culture of Care ist eine klare Hierarchie von Vorteil.

Stimme gar nicht zu	Stimme eher nicht zu	Teils, teils	Stimme eher zu	Stimme voll zu	Keine Antwort
☐	☐	☐	☐	☐	☐

Katharina Ameli & Stephanie Krämer
ICAR3R, Justus-Liebig-Universität Gießen

Frageblock zu individuellen Wahrnehmungen

28. Ich schätze die Tiere, mit denen ich arbeite.

Stimme gar nicht zu	Stimme eher nicht zu	Teils, teils	Stimme eher zu	Stimme voll zu	Keine Antwort
☐	☐	☐	☐	☐	☐

29. Ich blende während meiner Arbeit mit den Versuchstieren meine Gefühle und Emotionen weitgehend aus.

ⓘ Hinweis: Hier geht es um Emotionen, die sich im Arbeitskontext mit den Tieren ergeben.

Stimme gar nicht zu	Stimme eher nicht zu	Teils, teils	Stimme eher zu	Stimme voll zu	Keine Antwort
☐	☐	☐	☐	☐	☐

30. Die Tötung von Tieren macht mir zu schaffen.

Stimme gar nicht zu	Stimme eher nicht zu	Teils, teils	Stimme eher zu	Stimme voll zu	Keine Antwort
☐	☐	☐	☐	☐	☐

31. Ich fühle mich für ein gutes Miteinander im Team verantwortlich.

Stimme gar nicht zu	Stimme eher nicht zu	Teils, teils	Stimme eher zu	Stimme voll zu	Keine Antwort
☐	☐	☐	☐	☐	☐

32. Ich fühle mich für ein gutes Miteinander im Team verantwortlich.

Stimme gar nicht zu	Stimme eher nicht zu	Teils, teils	Stimme eher zu	Stimme voll zu	Keine Antwort
☐	☐	☐	☐	☐	☐

33. Ich fühle mich in der Lage Veränderungen anzustoßen.

Stimme gar nicht zu	Stimme eher nicht zu	Teils, teils	Stimme eher zu	Stimme voll zu	Keine Antwort
☐	☐	☐	☐	☐	☐

34. Bringe ich Veränderungsideen ein, werden diese umgesetzt.

Stimme gar nicht zu	Stimme eher nicht zu	Teils, teils	Stimme eher zu	Stimme voll zu	Keine Antwort
☐	☐	☐	☐	☐	☐

35. Ich werde in meiner Person und mit meinem Wissen ernst genommen.

Stimme gar nicht zu	Stimme eher nicht zu	Teils, teils	Stimme eher zu	Stimme voll zu	Keine Antwort
☐	☐	☐	☐	☐	☐

36. **Meine Institution bietet mir die Möglichkeit für regelmäßige, persönliche Weiterentwicklung.**
ⓘ Hinweis: Persönliche Weiterentwicklung schließt hier Fortbildungen, persönliche Gespräche, Coachings o. Ä. mit ein.

Stimme gar nicht zu	Stimme eher nicht zu	Teils, teils	Stimme eher zu	Stimme voll zu	Keine Antwort
☐	☐	☐	☐	☐	☐

37. Die Nutzung der Versuchstiere für den wissenschaftlichen Fortschritt ist alternativlos.

Stimme gar nicht zu	Stimme eher nicht zu	Teils, teils	Stimme eher zu	Stimme voll zu	Keine Antwort
☐	☐	☐	☐	☐	☐

Frageblock zum Wohlbefinden von Tieren

38. Die Verantwortlichkeit für das Wohlbefinden der Tiere liegt bei der…

	Stimme gar nicht zu	Stimme eher nicht zu	Teils, teils	Stimme eher zu	Stimme voll zu	Keine Antwort
Leitungsebene	☐	☐	☐	☐	☐	☐
Tierpflegerebene	☐	☐	☐	☐	☐	☐
Wissenschaftsebene	☐	☐	☐	☐	☐	☐
Überwachende Ebene	☐	☐	☐	☐	☐	☐

39. Die Verantwortlichkeit für das Wohlbefinden der Tiere liegt bei der/dem…

	Stimme gar nicht zu	Stimme eher nicht zu	Teils, teils	Stimme eher zu	Stimme voll zu	Keine Antwort
Institutsleiter*in	☐	☐	☐	☐	☐	☐
Tierhausleiter*in	☐	☐	☐	☐	☐	☐
Projektleitung	☐	☐	☐	☐	☐	☐
Tierpfleger*innen	☐	☐	☐	☐	☐	☐
Wissenschaftliche Leitung	☐	☐	☐	☐	☐	☐
Postdoktorand*innen und Doktorand*innen	☐	☐	☐	☐	☐	☐
Behörde	☐	☐	☐	☐	☐	☐
Tierschutzbeauftragte	☐	☐	☐	☐	☐	☐
Tierärztliche Leitung	☐	☐	☐	☐	☐	☐

Frageblock zum Wohlbefinden von Mitarbeitenden

40. Die Verantwortlichkeit für das Wohlbefinden der Mitarbeiter*innen liegt bei der…

	Stimme gar nicht zu	Stimme eher nicht zu	Teils, teils	Stimme eher zu	Stimme voll zu	Keine Antwort
Leitungsebene	☐	☐	☐	☐	☐	☐
Tierpflegerebene	☐	☐	☐	☐	☐	☐
Wissenschaftsebene	☐	☐	☐	☐	☐	☐
Überwachende Ebene	☐	☐	☐	☐	☐	☐

41. Die Verantwortlichkeit für das Wohlbefinden der Mitarbeiter*innen liegt bei der/dem…

	Stimme gar nicht zu	Stimme eher nicht zu	Teils, teils	Stimme eher zu	Stimme voll zu	Keine Antwort
Institutsleiter*in	☐	☐	☐	☐	☐	☐
Tierhausleiter*in	☐	☐	☐	☐	☐	☐
Projektleitung	☐	☐	☐	☐	☐	☐
Tierpfleger*innen	☐	☐	☐	☐	☐	☐
Wissenschaftliche Leitung	☐	☐	☐	☐	☐	☐
Postdoktorand*innen und Doktorand*innen	☐	☐	☐	☐	☐	☐
Behörde	☐	☐	☐	☐	☐	☐
Tierschutzbeauftragte	☐	☐	☐	☐	☐	☐
Tierärztliche Leitung	☐	☐	☐	☐	☐	☐

Frageblock zur Kommunikation zwischen den Ebenen

42. Die Kommunikation/Austausch zwischen den folgenden Ebenen wird praktiziert.

ⓘ **Leitungsebene** (Institutsleiter*in, Tierhausleiter*in oder tierärztliche Leitung)
Wissenschaftsebene (wiss. Leitung, Postdoktorand*innen und Doktorand*innen oder Projektleitung)
Überwachende Ebene (Zuständige Behörde = Behörde, Tierschutzbeauftragte)

	Stimme gar nicht zu	Stimme eher nicht zu	Teils, teils	Stimme eher zu	Stimme voll zu	Keine Antwort
**Leitungsebene und Tierpfleger*innen**	☐	☐	☐	☐	☐	☐
**Leitungsebene und überwachende Ebene**	☐	☐	☐	☐	☐	☐
**Leitungsebene und Wissenschaftsebene**	☐	☐	☐	☐	☐	☐
**Leitungsebene und Tierschutzbeauftragte**	☐	☐	☐	☐	☐	☐
**Wissenschaftsebene und überwachende Ebene**	☐	☐	☐	☐	☐	☐
**Wissenschaftsebene und Tierpfleger*innen**	☐	☐	☐	☐	☐	☐
**Wissenschaftsebene und Tierschutzbeauftragte**	☐	☐	☐	☐	☐	☐
**Überwachende Ebene und Tierpfleger*innen**	☐	☐	☐	☐	☐	☐
**Überwachende Ebene und Tierschutzbeauftragte**	☐	☐	☐	☐	☐	☐
**Tierpfleger*innen und Tierschutzbeauftragte**	☐	☐	☐	☐	☐	☐
**Wissenschaftsebene und Wissenschaftsebene**	☐	☐	☐	☐	☐	☐
**Tierpfleger*innen und Tierpfleger*innen**	☐	☐	☐	☐	☐	☐
**Leitungsebene und Leitungsebene**	☐	☐	☐	☐	☐	☐
**Tierschutzbeauftragte und Tierschutzbeauftragte**	☐	☐	☐	☐	☐	☐

Vielen Dank für Ihre Teilnahme!
